# Modelling sentiments based on objectivity and subjectivity with self-attention mechanisms

**DOI:** 10.12688/f1000research.73131.1

**Published:** 2021-10-04

**Authors:** Hu Ng, Glenn Jun Weng Chia, Timothy Tzen Vun Yap, Vik Tor Goh

**Affiliations:** 1 Faculty of Computing and Informatics, Multimedia University, Cyberjaya, Selangor, 63100, Malaysia; 2 Faculty of Engineering, Multimedia University, Cyberjaya, Selangor, 63100, Malaysia

**Keywords:** Sentiment analysis, subjectivity, objectivity, attention mechanism, neural nets.

## Abstract

**Background** **:** The proliferation of digital commerce has allowed merchants to reach out to a wider customer base, prompting a study of customer reviews to gauge service and product quality through sentiment analysis. Sentiment analysis can be enhanced through subjectivity and objectivity classification with attention mechanisms.

**Methods**: This research includes input corpora of contrasting levels of subjectivity and objectivity from different databases to perform sentiment analysis on user reviews, incorporating attention mechanisms at the aspect level. Three large corpora are chosen as the subjectivity and objectivity datasets, the Shopee user review dataset (ShopeeRD) for subjectivity, together with the Wikipedia English dataset (Wiki-en) and Internet Movie Database (IMDb) for objectivity. Word embeddings are created using Word2Vec with Skip-Gram. Then, a bidirectional LSTM with an attention layer (LSTM-ATT) imposed on word vectors. The performance of the model is evaluated and benchmarked against classification models of Logistics Regression (LR) and Linear SVC (L-SVC). Three models are trained with subjectivity (70% of ShopeeRD) and the objectivity (Wiki-en) embeddings, with ten-fold cross-validation. Next, the three models are evaluated against two datasets (IMDb and 20% of ShopeeRD). The experiments are based on benchmark comparisons, embedding comparison and model comparison with 70-10-20 train-validation-test splits. Data augmentation using AUG-BERT is performed and selected models incorporating AUG-BERT, are compared.

**Results:** L-SVC scored the highest accuracy with 56.9% for objective embeddings (Wiki-en) while the LSTM-ATT scored 69.0% on subjective embeddings (ShopeeRD).  Improved performances were observed with data augmentation using AUG-BERT, where the LSTM-ATT+AUG-BERT model scored the highest accuracy at 60.0% for objective embeddings and 70.0% for subjective embeddings, compared to 57% (objective) and 69% (subjective) for L-SVC+AUG-BERT, and 56% (objective) and 68% (subjective) for L-SVC.

**Conclusions**: Utilizing attention layers with subjectivity and objectivity notions has shown improvement to the accuracy of sentiment analysis models.

## Introduction

The proliferation of digital commerce, especially in Malaysia, has allowed many local merchants to reach out to a wider customer base. In order to attract customer’s attention, merchants always compete to offer better price and higher quality of services. Besides that, they also seriously consider the customer feedback or reviews in order to gauge service and product quality. ^1^

By exploring the sentiment tendency of customer reviews, it can provide a good reference for other customer before the purchasing decision is made. Besides, it helps merchants to improve service quality and customer satisfaction.

Sentiment analysis is aimed to determine the sentiment as well as polarity on part of a text. Normally, language terms are under two form of statements, namely fact statement and a non-fact statement, which are known as objective and subjective in categorical terms. ^2^ Facts are objective terms likes events entities and their properties. On the other hand, a non-fact statement is subjective and usually related to an individual’s sentiments, personal beliefs, opinion, perspective, feelings or thoughts.

This paper adopted attention segment ^3^ to a neural network, LSTM, by creating attention-weighted features, namely Long Short Term Memory with Attention (LSTM-ATT) ^4^ to create attention-weighted features. It aims to introduce these features at the input level to the neural network, so that the performance of sentiment can be increased.

In business operations, sentiment classification is the automated process of classifying customer reviews in text and labeling them as positive, negative, or neutral. It employs Natural Language Processing (NLP) routine to interpret subjective data, so that it can help merchants to understand how customers feel about their products or services. In this paper, sentiment classification is performed by non-deep neural network classifiers, namely Logistic Regression (LR) and Linear SVC (L-SVC) together with Deep Learning (Artificial Neural Networks) classifier by adopting attention mechanisms (LSTM-ATT).

### Word embedding

Word embeddings are a scheme to convert human language to a word representation that is understandable by computers. The word representation is in the form of a real-valued vector that encodes the meaning of the word, so that the words that are closer in the vector space are expected to be similar in meaning.

Collobert ^5^ declared that a distinction word vector and proper training can increase the performance of NLP works especially the sentiment analysis. Word embedding can be classified into two types; contextual and non-contextual embeddings. Non-contextual embedding does not consider the effects of arrangement of words in a particular sentence, while contextual embedding does the opposite.

For non-contextual embedding, Mikolov *et al*. initiated Word2Vec. ^6^ The word2vec algorithm uses a neural network model to learn word associations from a large corpus of text. Once trained, such a model can detect synonymous words or suggest additional words for a partial sentence. Bengio *et al.* ^7^ and Collobert *et al.* ^8^ enhanced it by implementing Neutral Net Language Model (NNLM). Bojanowski *et al*. ^9^ made enhancement on Word2Vec by applying n-grams and cables to obtain higher performance in Word Similarity assignments that involved various types of languages and was able to show big enhancements on morphology rich languages, in particular, German datasets such as GUR350 and GUR65 ^10^ and ZG222. ^11^ Bhagat *et al*. ^12^ applied unigrams to extra individual words from Twitter messages and multiple machine learning techniques to perform sentiment analysis. Ebner *et al.* ^13^ employed three simple bag-of-words representations, where a text is represented as the bag (multiset) of its words, namely pooling encoders, pre-trained word embeddings, and unigram generative regularization to regularize incorporating auxiliary discriminative tasks that managed to reduce training time and model size while maintaining high performance. Gayatry ^14^ employed Count Vectorizer to convert each word into its corresponding vectors.

For context embedding, Peters *et al.* ^15^ modified LSTM neural nets to create Embedding from Language Models (ELMo) that were able to show better results than the Stanford Tree-bank model (SST-5) from the research work by Socher *et al.* ^16^ Devlin *et al.* ^17^ constructed BERT along with Transformers and Attention Mechanism. ^3^ The role of BERT is not limited to embedding functions but also become a language model that is capable to exceed ELMo on General Language Understanding Evaluation assignments (GLUE) from the research outcomes by Wang *et al.* ^18^ Liu *et al.* ^19^ enhanced BERT by developing A Robustly Optimized BERT Pre-Training Approach (RoBERTa). RoBERTa omits the Next Sentence Prediction task and applies an unfixed masking configuration rather than static Masked Language Modelling (MLM). Sangeetha ^20^ proposed multi-head attention fusion model of word and context embedding for sentiment analysis of student feedback.

## Methods

### Ethics approval

Ethical Approval Number: EA1602021 (From Technology Transfer Office (TTO), Multimedia University).

### Datasets

Three large corpora datasets were chosen to denote objectivity and subjectivity datasets correspondingly. IMDb ^21^ and Wiki-en ^22^ were chosen as the objectivity datasets while ShopeeRD ^23^ was chosen as the subjectivity dataset.

IMDb consists of 50K of movie reviews with contents based on the true plot and written with a neutral point of view (NPOV). Wiki-en consists of 4677K of records based on Wikipedia that forced the articles to be factual and follow the NPOV policy. ShopeeRD consists of 208K customer reviews taken from the Shopee Code League 2020 Data Science and Data Analytics competition. ShopeeRD’s entries are based on customer experiences, which are potentially judgemental and opinionated.

**Figure 1.**
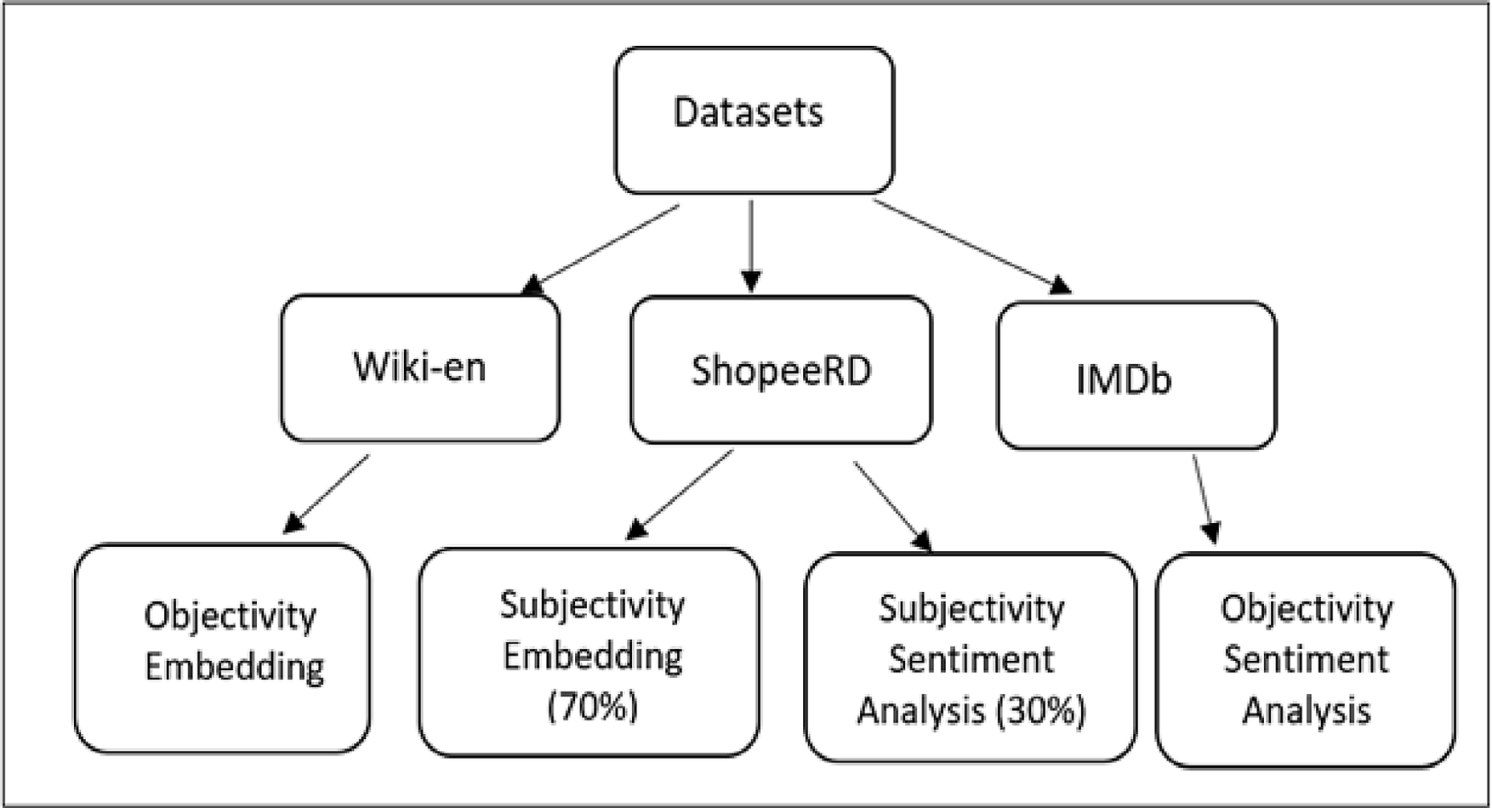
The mapping of datasets.

Wiki-en was used as the objectivity corpus for word embedding, while IMDb was used for objectivity sentiment analysis. 70% of the ShopeeRD was used as the subjectivity corpus for word embedding and the remaining 30% for subjectivity sentiment analysis. Figure 1 displays the mapping of datasets.

### Data preparations and word embedding

The reviews and records from the datasets underwent a set of data cleaning steps which included emoji cleaning, text cleaning such as repeated character elimination, punctuation (e.g., ?, ! or,) elimination, stop word (e.g., becomes, against, or at) elimination, lemmatization, case lowering and normalization (normalizing non-English writing into English writing).

Word embedding is carried out to transform the reviews into floating-point numbers that are stored in a high dimension array, which forms a dictionary that the computer is able to obtain word vectors from. The word embedding must be large enough to represent millions of words and for each word is denoted as a high dimension vector. In this paper, one word is represented as a 300-dimension vector.

Word2Vec by Mikolov et al. ^24^ is a word embedding method that consist of two structural design, namely Skip-gram and Continuous Bag-of-Words (CBOW). In the CBOW model, the distributed representations of context (or surrounding words) are combined to predict the word in the middle, while in the Skip-gram model, the distributed representation of the input word is used to predict the context. It has been proven that the Skip-gram structure has been shown better results in comparison to Continuous Bag-of-Words. ^25- 27^ Hence, this paper utilizes Word2Vec Skip-Gramm structure to perform word embedding.

ShopeeRD and Wiki-en were trained into embeddings of 300d (300-dimension), with a factor of five negative examples, window dimension of five tokens, and elimination of small sentences. The two embeddings (subjectivity and objectivity) were trained for ten repetitions.

### Models

To prevent over-fitting or one model favouring towards a particular embedding, two models (LR and L-SVC) were applied for this paper. In general, a sentence vector is produced from the formation of word vectors. ^28^ Nevertheless, this paper assumes that certain letterings might not apply any weight or produce any consequence, therefore an attention layer, which is adopted from Vaswani *et al.* ^3^ was produced as a substitute. Self-attention is capable of allocating ‘attention’ to an important vector (keyword). This permits the structural design in a way to highlight attention-ed vectors. ^29^

**Figure 2.**
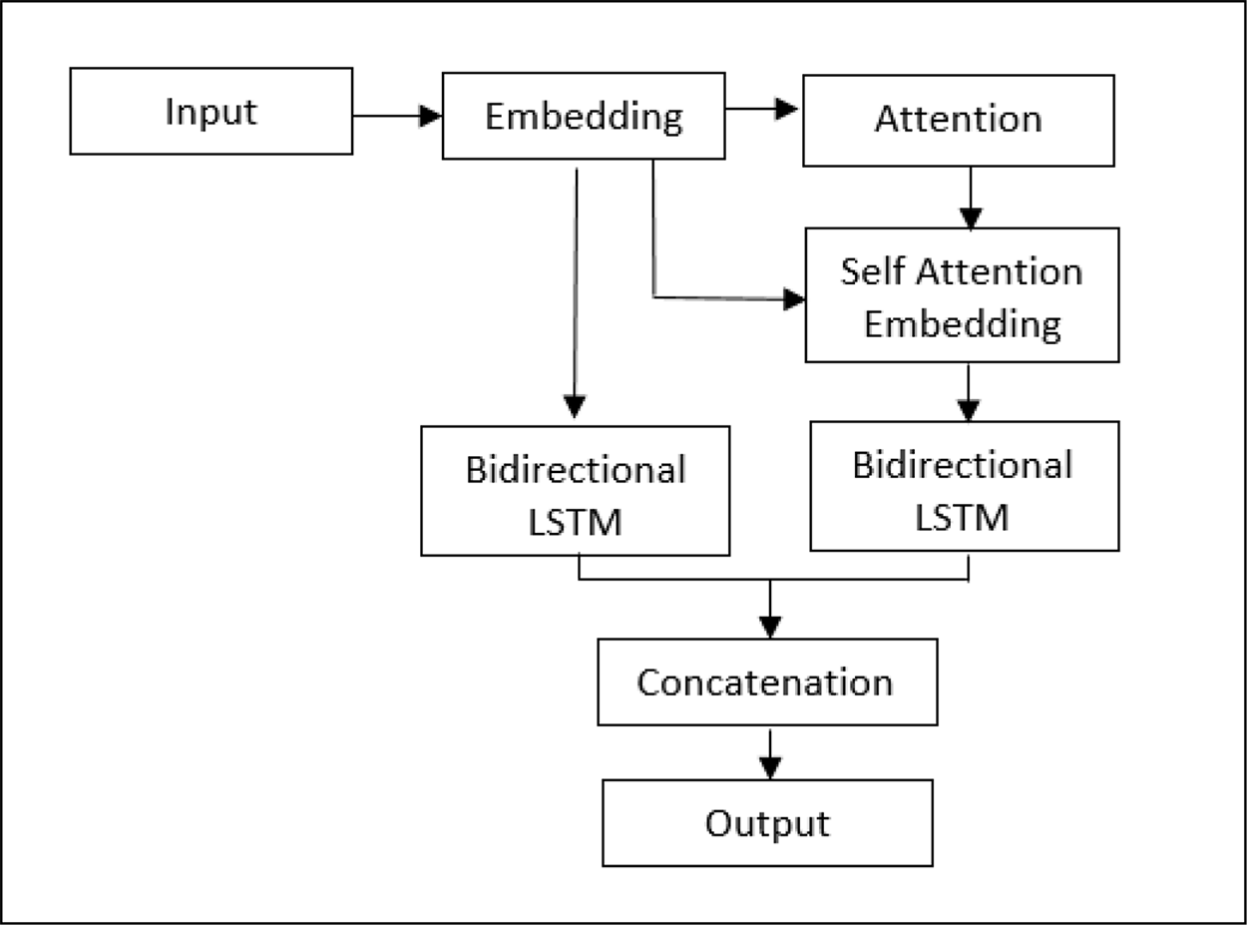
Structural design of LSTM-ATT.

For that reason, a model integrating attention segments was recommended, and the structural design is presented in Figure 2. The word vectors worked through the attention layer, creating attention-weighted features. By adapting LSTM neural nets, both the original embedding and the attention-weighted embedding are concatenated to create sentiment features.

**Figure 3.**
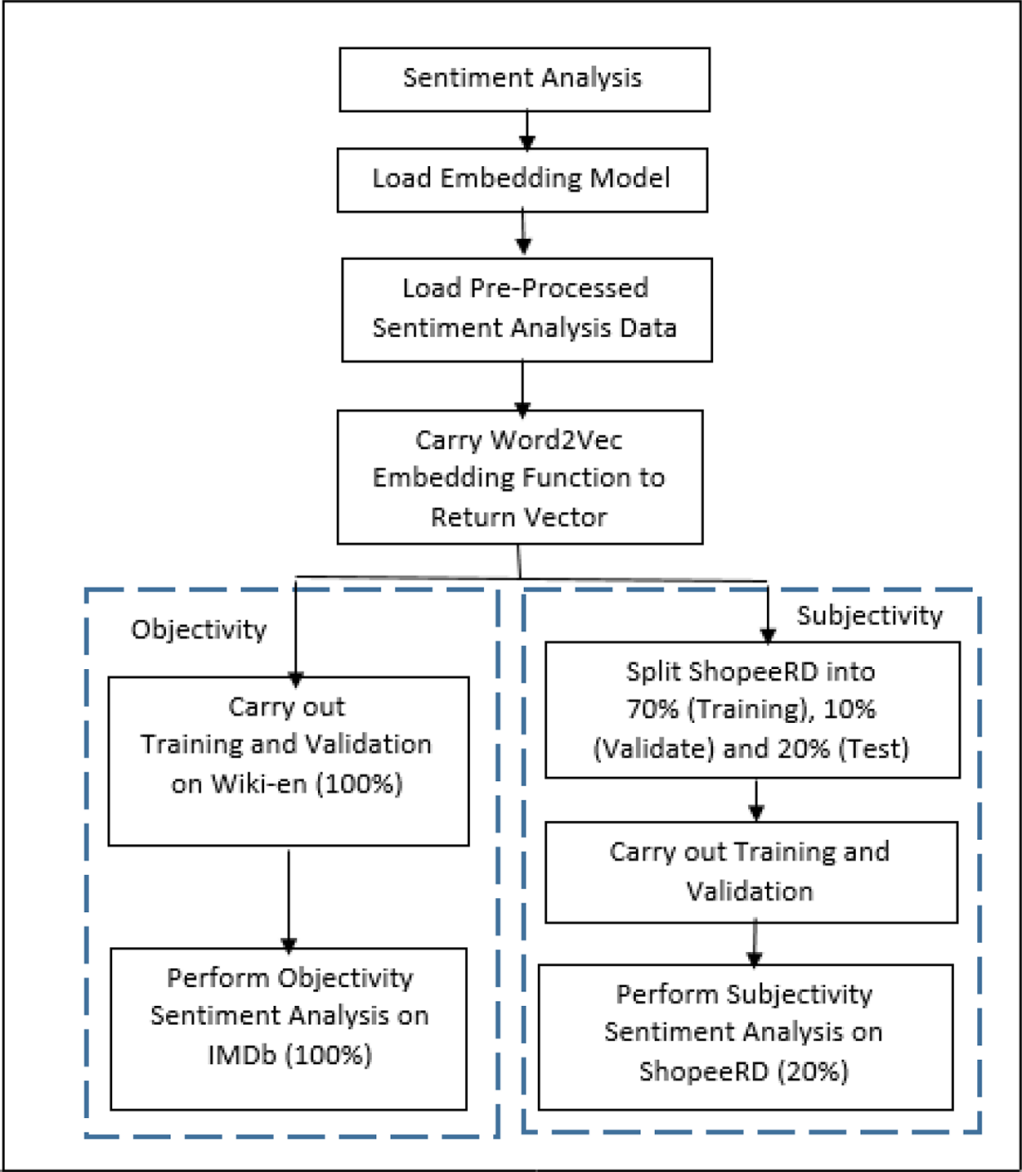
Sentiment analysis.

This paper adopted attention-weighted features model is called Long Short Term Memory with Attention (LSTM-ATT) ^4^ with intention to improve the sentiment performance. These features at the input level to the neural network and go through a few dense layers to flatten the output. Finally, Rectified Linear Unit (RELu), a non-linear activation function is applied to produce the sentiment results. The model, LSTM-ATT, is then evaluated against LR and L-SVC. The workflow of the sentiment analysis on IMDb and ShopeeRD with multiple models is illustrated in Figure 3.

## Results and discussion

### Design of experiments

The experiments were performed in Python, utilizing the scikit-learn library for machine learning as well as the BERT model architecture. Three models (LR, L-SVC and LSTM-ATT) were trained with the objectivity (Wiki-en) and subjectivity (70% of ShopeeRD) embeddings. Ten-fold cross-validation was applied during the training. After that, the models were tested against the objectivity (IMDb) test set and the subjectivity (20% of ShopeeRD) test set to eliminate bias.

The experiments were based on benchmark comparison, embedding comparison and model comparison with 70-10-20 train-validation-test splits. The validation was carried out to perform parameter tuning, so that the best results among the models could be obtained.

### Quality of embeddings

**Figure 4.**
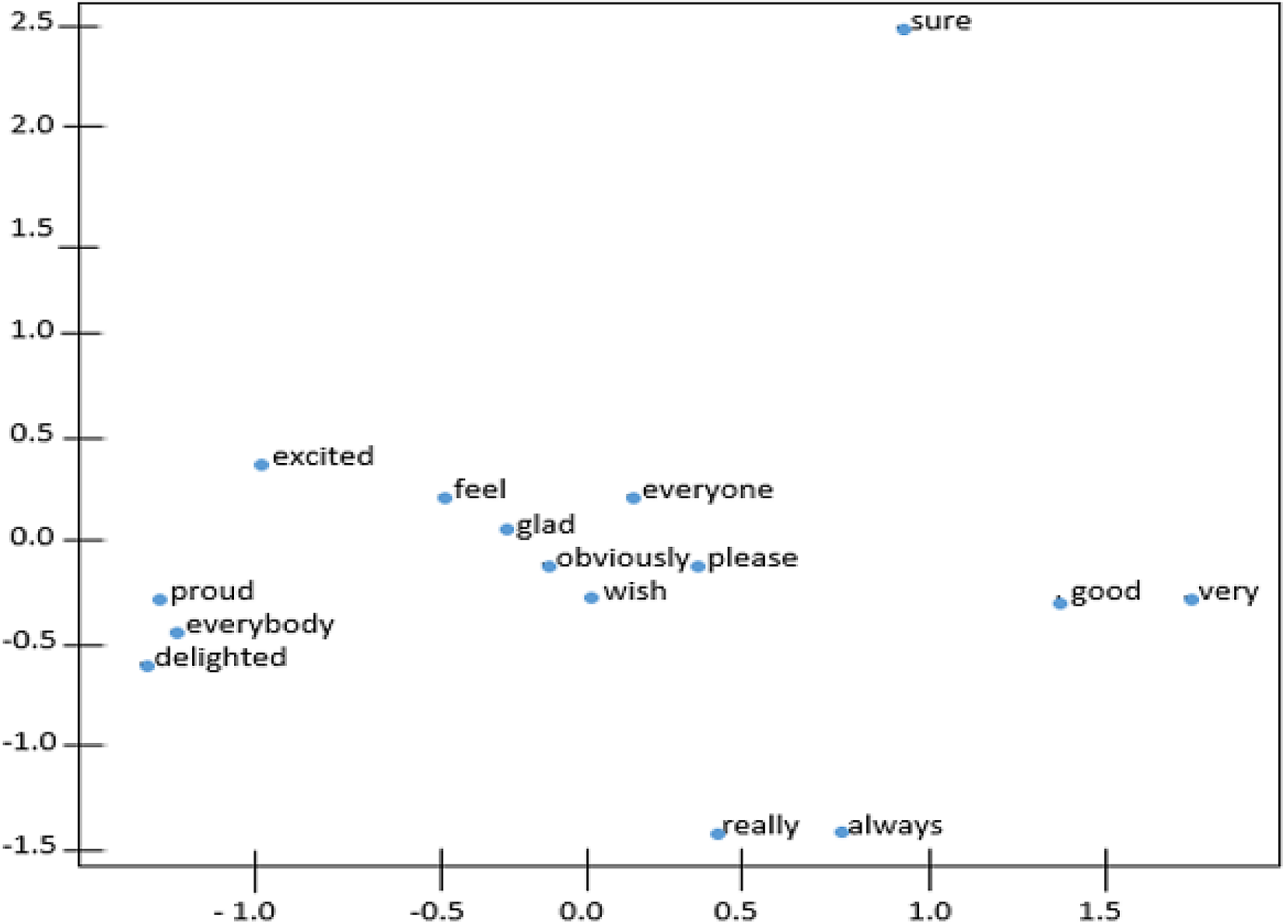
t-SNE plots of ‘happy’ on the Wiki-en.

**Figure 5.**
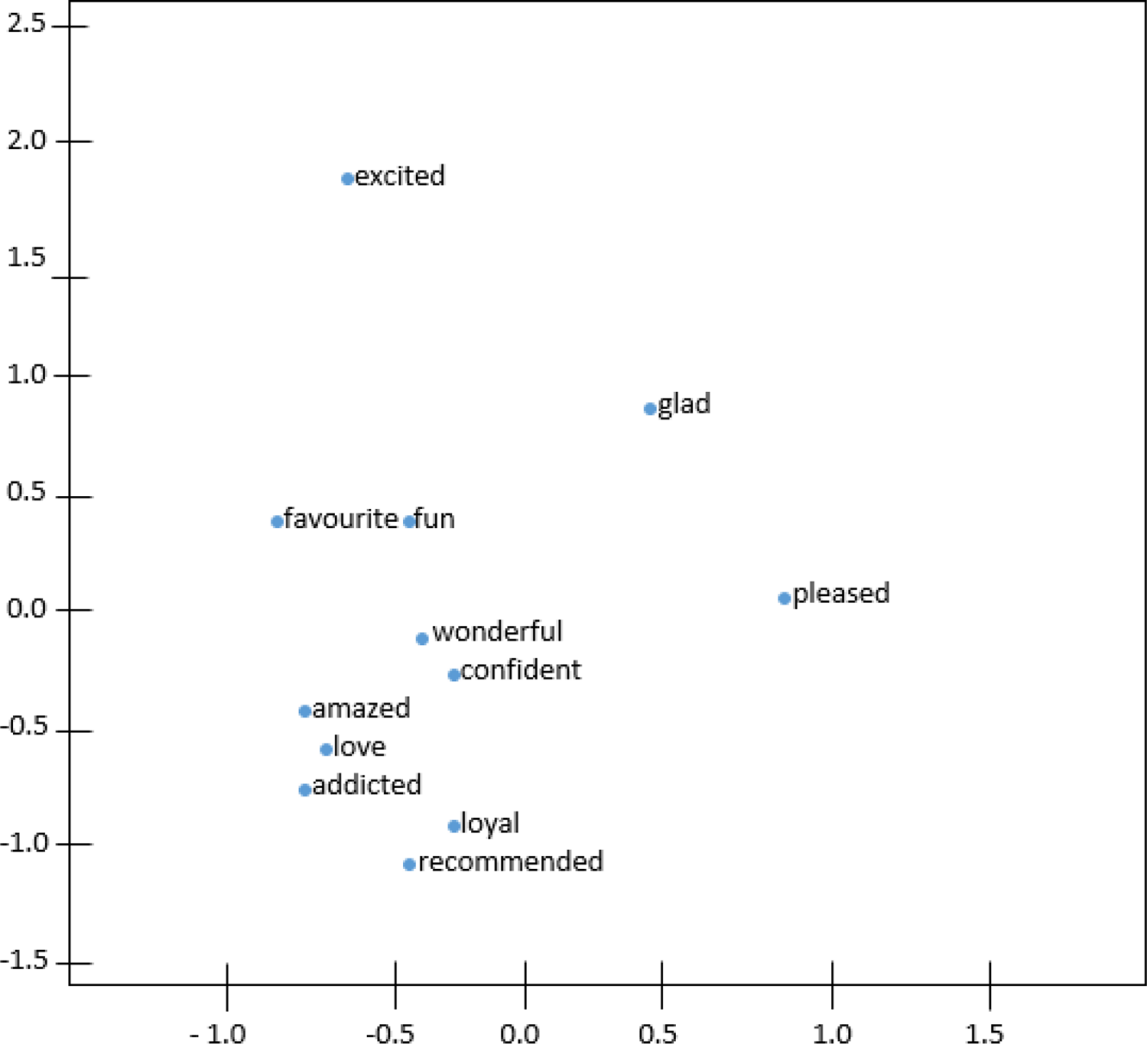
t-SNE plots of ‘happy’ on the ShopeeRD.

Figures 4 and 5 demonstrate the t-distributed Stochastic Neighbor Embedding (t-SNE) plots for Wiki-en and ShopeeRD embeddings on the top 15 nearest words to the word ‘happy’. The t-SNE for both datasets revealed that word similarities are discovered in the embeddings, for instance, ‘glad’, ‘pleased’, ‘excited’ are grouped together with ‘happy’.

The words ‘very’ and ‘good’ having closeness to ‘happy’ were only found in t-SNE for Wiki-en only. Meanwhile the words ‘satisfied’ and ‘wonderful’ having closeness to ‘happy’ were found in t-SNE for ShopeeRD. Furthermore, outliers like ‘everyone’ and ‘everybody’ were found to appear in the t-SNE for Wiki-en. This shows that the two embeddings are different in nature.

### Sentiment analysis

The three models namely LR, L-SVC and LSTM-ATT were evaluated in terms of their performance in sentiment analysis. The accuracy of the three models is presented in Table 1. L-SVC obtained the highest accuracy (56.9%) for objectivity embedding, whereas LSTM-ATT obtained the highest accuracy (69.0%) for subjectivity embedding. L-SVC performed better than LR properly due to L-SVC attempting to exploit the margin between the closest support vectors whereas LR exploits the posterior class probability. ^30^

**Table 1.** Accuracy of three classifiers.

Data	Model		
	LR	L-SVC	LSTM-ATT
Objective embedding	0.5338	0.5685	0.5604
Subjective embedding	0.6418	0.6892	0.6902

From Table 1, there is possible limitation factor cause by the capacity of the training data, therefore the size of the dataset is increased through the data augmentation technique. ^31^ As LR has a simpler architecture, data augmentation is not considered, and the focus is made on L-SVC and LSTM-ATT. Table 2 presents the outcome of data augmentation.

**Table 2.** Accuracy of two classifiers with augmentation technique.

Data	Model	
	L-SVC+AUG-BERT	LSTM-ATT+ AUG-BERT
Objective embedding	0.5746	0.5991
Subjective embedding	0.6907	0.7004

From Table 2, it is found that the accuracy of models with the augmented data are found to be better than the models, although not by much. The LSTM-ATT+AUG-BERT was able to beat L-SVC+AUG-BERT on both objective and subjective embeddings.

To the best of our knowledge, there is only one sentiment analysis result from Gayatry’s work ^14^ that accepted by Shopee Code League 2020 Data Science. ^32^ Table 3. shows the comparison of our models with Gayatry’s work on ShopeeRD.

**Table 3.** Comparison of our models with others research work on ShopeeRD.

Data	Model		
ShopeeRD	L-SVC+AUG-BERT [our method]	LSTM-ATT+AUG-BERT [our method]	Multinomial Naïve Bayes ^14^
Training (110K), Testing (36K)	-	-	0.58
Training (145K), Testing (41K)	0.69	0.70	-

To the best of our knowledge, there is no research work on objectivity sentiment analysis on IMDB without any involvement of pre-train data from taken IMDB, as all of them used 50% of total dataset for training and another 50% for testing. In this paper, we trained the models by Wiki-en and test on IMDB.

## Conclusions

This paper has presented word embeddings for both objectivity and subjectivity contexts by applying Word2Vec. Analyzing the embedding using the t-distributed stochastic neighbour embedding plot shows that there are some similarities between the two embeddings，but a majority of them are dissimilar. Three models namely, LR, L-SVC and LSTM-ATT were employed to evaluate the performance of the adopted embedding technique. The attention model adopted was able to perform sentiment analysis well with the requirement of more data was fed into the model utilizing AUG-BERT data augmentation. Models with differing architectures will be explored in future work.

## Data availability

### Underlying data

- Compiled Movie reviews from the Internet Movie Database (IMDb) : https://datasets.imdbws.com/, ^21^ cited on 6 August 2021.

The data are available for personal and non-commercial use, as stipulated by the owner (IMDb).

- A complete copy of all Wikimedia wikis, in the form of wikitext source and metadata embedded in XML: https://dumps.wikimedia.org/backup-index.html, ^22^ cited on 6 August 2021.

The data are available under the terms of the Creative Commons Attribution-Share-Alike 3.0 License.

- Product reviews from the Shopee e-commerce platform, created for the Shopee Code League 2020 Data Science and Data Analytics competitions: https://www.kaggle.com/davydev/shopee-code-league-20, ^23^ cited on 6 August 2021.

The data are available for personal and non-commercial use, as stipulated by the owner (Shopee).
