# Supplementary material for: Modelling sentiments based on objectivity and subjectivity with self-attention mechanisms
Source: F1000Res. 2022 May 17;10:1001. Originally published 2021 Oct 4. [Version 2] doi: 10.12688/f1000research.73131.2 (PMC9130759; doi:10.12688/f1000research.73131.2)
Supplement: Peer review history

# Peer review: Reviewer response for version 2

**DOI:** 10.5256/f1000research.134045.r138119

**Published:** 2022-05-24

**Authors:** Hien D. Nguyen

**Affiliations:** 1 University of Information Technology, VNU-HCM, Ho Chi Minh City, Vietnam

This paper revised as my recommendation. In my opinion, this paper can be indexed.

Is the work clearly and accurately presented and does it cite the current literature?

Partly

If applicable, is the statistical analysis and its interpretation appropriate?

Yes

Are all the source data underlying the results available to ensure full reproducibility?

Yes

Is the study design appropriate and is the work technically sound?

Yes

Are the conclusions drawn adequately supported by the results?

Yes

Are sufficient details of methods and analysis provided to allow replication by others?

Yes

Reviewer Expertise:

Intelligent system, knowledge engineering, data science, automated reasoning, machine learning

I confirm that I have read this submission and believe that I have an appropriate level of expertise to confirm that it is of an acceptable scientific standard.

# Peer review: Reviewer response for version 1

**DOI:** 10.5256/f1000research.76757.r126996

**Published:** 2022-04-08

**Authors:** Marco Polignano

**Affiliations:** 1 Department of Computer Science, University of Bari 'Aldo Moro', Bari, Italy

The authors present an approach to performing sentiment analysis by comparing different embeddings and classification strategies. 

 Several weaknesses need to be addressed by the authors to enable publication:

1. Insufficient detail is provided on the motivations for the article. Why is what is presented relevant to scientific research? 
2. Numerous approaches in the literature use an attention level LSTM model to do sentiment analysis. What does this work present that is innovative?
3. Technical and implementation details are not provided. Source code is not shared. This makes the proposed contribution non-replicable.
4. It is unclear why non-contextual embeddings were used and not the newer ones based on BERT and ELMO. It is unclear how the data is used in the two phases of embedding space creation and sentiment analysis. Further details on the experimental protocol should be reported.
5. The authors show accuracy as the only metric. This metric is not applicable if the datasets are unbalanced. Unfortunately, such analysis is not performed by the authors. I suggest including the scores of additional metrics such as precision, recall, and f1 measure.
6. A statistical validation of the results has not been conducted, so the differences obtained are not supported by real-world evidence and may be due to chance.

Therefore, it is suggested to further investigate these issues to make the contribution more robust and relevant to the community.

Is the work clearly and accurately presented and does it cite the current literature?

Partly

If applicable, is the statistical analysis and its interpretation appropriate?

Not applicable

Are all the source data underlying the results available to ensure full reproducibility?

Yes

Is the study design appropriate and is the work technically sound?

Partly

Are the conclusions drawn adequately supported by the results?

Partly

Are sufficient details of methods and analysis provided to allow replication by others?

No

Reviewer Expertise:

Natural Language Processing, Machine Learning, Sentiment Analysis, Recommender Systems

I confirm that I have read this submission and believe that I have an appropriate level of expertise to confirm that it is of an acceptable scientific standard, however I have significant reservations, as outlined above.

## References

[1] : A comparison of word-embeddings in emotion detection from text using bilstm, cnn and self-attention. *Adjunct Publication of the 27th Conference on User Modeling, Adaptation and Personalization* .2019;63-68

[2] : Alberto: Italian BERT language understanding model for NLP challenging tasks based on tweets. *6th Italian Conference on Computational Linguistics, CLiC-it 2019* .2019;2481:1-6

[3] : Do You Feel Blue? Detection of Negative Feeling from Social Media.10640: 10.1007/978-3-319-70169-1_24 321-333 10.1007/978-3-319-70169-1_24

# Peer review: Reviewer response for version 1

**DOI:** 10.5256/f1000research.76757.r126992

**Published:** 2022-03-23

**Authors:** Hien D. Nguyen

**Affiliations:** 1 University of Information Technology, VNU-HCM, Ho Chi Minh City, Vietnam

This paper introduced an architecture to adopt attention segment to a neural network, called LSTM-ATT, to create attention-weighted features. Through that, sentiment classification is performed by non-deep neural network classifiers, this method is called Logistic Regression (LR) and Linear SVC (L-SVC) together with Deep Learning classifier by LSTM-ATT. The results show that L-SVC scored the highest accuracy with 56.9% for objective embeddings (Wiki-en) and LSTM-ATT scored 69.0% on subjective embeddings. The authors also experimented by integrating the proposed method with data augmentation using AUG-BERT. The LSTM-ATT+AUGBERT model scored the highest accuracy at 60.0% for objective embeddings and 70.0% for subjective embeddings, and  57% (objective) and 69% (subjective) for L-SVC+AUG-BERT.

 This paper is well organized. However, the authors should revise as follows:

1. The authors should explain more the importance about modeling sentiment based on objectivity and subjectivity.
2. This study has to present more the architectures in Figure 2 and Figure 3.
3. The results need to be explained more their meaning. The authors should compare the proposed method with other similar methods, such as:
  Nguyen, H., Huynh, T., Hoang, S., et al. (2020). Language-oriented Sentiment Analysis based on the Grammar Structure and Improved Self-attention Network. ENASE 2020 *,* pp. 339-346 ^1^
  Zainuddin, N., Selamat, A., Ibrahim, R. (2018). Hybrid sentiment classification on twitter aspect-based sentiment analysis. Applied Intelligence 48(5): 1218 – 1232 ^2^
4. The references [14] and [32] should be added their titles.

In my opinion, this paper need to be revised before the final decision.

Is the work clearly and accurately presented and does it cite the current literature?

Partly

If applicable, is the statistical analysis and its interpretation appropriate?

Yes

Are all the source data underlying the results available to ensure full reproducibility?

Yes

Is the study design appropriate and is the work technically sound?

Yes

Are the conclusions drawn adequately supported by the results?

Yes

Are sufficient details of methods and analysis provided to allow replication by others?

Yes

Reviewer Expertise:

Intelligent system, knowledge engineering, data science, automated reasoning, machine learning

I confirm that I have read this submission and believe that I have an appropriate level of expertise to confirm that it is of an acceptable scientific standard, however I have significant reservations, as outlined above.

## References

[1] : Language-oriented Sentiment Analysis based on the Grammar Structure and Improved Self-attention Network. *Proceedings of the 15th International Conference on Evaluation of Novel Approaches to Software Engineering (ENASE 2020)* .2020; 10.5220/0009358803390346 339-346 10.5220/0009358803390346

[2] : Hybrid sentiment classification on twitter aspect-based sentiment analysis. *Applied Intelligence* .2017; 10.1007/s10489-017-1098-6 10.1007/s10489-017-1098-6

# Response

**Published:** 2022-05-13

**Authors:** Hu NG

**Affiliations:** Multimedia University, Malaysia

Dear Prof Nguyen,

 We are greatly appreciative of the insightful comments and helpful suggestions that you have provided.

 The following are our response on the issues that you have highlighted:

 Your Comment

 1. The authors should explain more the importance about modeling sentiment based on objectivity and subjectivity.

 Our Response

 We are of the opinion that once subjectivity is identified the sentiment accuracy can be further increased as objective statements should not contribute to this.

 Your Comment

 2.This study has to present more the architectures in Figure 2 and Figure 3.

 Our Response

 The references have also been corrected.

 Your Comment

 3.The results need to be explained more their meaning. The authors should compare the proposed method with other similar methods, such as:

 Nguyen, H., Huynh, T., Hoang, S., et al. (2020). Language-oriented Sentiment Analysis based on the Grammar Structure and Improved Self-attention Network. ENASE 2020, pp. 339-3461

 Zainuddin, N., Selamat, A., Ibrahim, R. (2018). Hybrid sentiment classification on twitter aspect-based sentiment analysis. Applied Intelligence 48(5): 1218 – 12322

 Our Response

 As we are using different datasets, we could not compare the results as is. However, we will perform this in future work. In addition, we take these papers into consideration and have cited them in the paper.

 Nguyen, H. D., Huynh, T., Hoang, S. N., Pham, V. T., & Zelinka, I. (2020, May). Language-oriented Sentiment Analysis based on the Grammar Structure and Improved Self-attention Network. In *ENASE* (pp. 339-346).

 4. The references [14] and [32] should be added their titles.

 Our Response

 The references have also been corrected.

# Peer review: Reviewer response for version 1

**DOI:** 10.5256/f1000research.76757.r123733

**Published:** 2022-02-28

**Authors:** Ashima Yadav

**Affiliations:** 1 Bennett University, Greater Noida, Uttar Pradesh, India

This paper has presented word embeddings for both objectivity and subjectivity contexts by applying Word2Vec. Analyzing the embedding using the t-distributed stochastic neighbour embedding plot shows that there are some similarities between the two embeddings, but a majority of them are dissimilar. Three models namely, LR, L-SVC and LSTM-ATT were employed to evaluate the performance of the adopted embedding technique. The attention model adopted was able to perform sentiment analysis well with the requirement of more data was fed into the model utilizing AUG-BERT data augmentation. The authors have very well addressed the problem. However, I still have following suggestions for them:

1. The authors should give a more detailed description of the deep learning based methods used in sentiment analysis. (Sentiment analysis using deep learning architectures: a review ^1^) (A Weighted Text Representation framework for Sentiment Analysis of Medical Drug Reviews ^2^)
2. Also, they should tell how their proposed model is different from the other baseline models (apart from giving higher accuracy) as the area of sentiment classification have many popular models.
3. Explain the role of attention mechanism in sentiment analysis. Can word-level and sentence level attention give better accuracy as explained in (A Language-independent Network to Analyze the Impact of COVID-19 on the World via Sentiment Analysis ^3^)?
4. In the Introduction section, a bullet-wise summary of the significant contribution of this work is required, which could highlight the motivation for this study.
5. There should be a Related work section in the manuscript which should explain how their proposed model differs from baseline methods or the advantages of their model over the baseline ones.

Is the work clearly and accurately presented and does it cite the current literature?

Yes

If applicable, is the statistical analysis and its interpretation appropriate?

Partly

Are all the source data underlying the results available to ensure full reproducibility?

Yes

Is the study design appropriate and is the work technically sound?

Yes

Are the conclusions drawn adequately supported by the results?

Partly

Are sufficient details of methods and analysis provided to allow replication by others?

Partly

Reviewer Expertise:

Sentiment analysis, deep learning, machine learning, attention mechanism

I confirm that I have read this submission and believe that I have an appropriate level of expertise to confirm that it is of an acceptable scientific standard, however I have significant reservations, as outlined above.

## References

[1] : Sentiment analysis using deep learning architectures: a review. *Artificial Intelligence Review* .2020;53(6) : 10.1007/s10462-019-09794-5 4335-4385 10.1007/s10462-019-09794-5

[2] : A Weighted Text Representation framework for Sentiment Analysis of Medical Drug Reviews. *2020 IEEE Sixth International Conference on Multimedia Big Data (BigMM)* .2020; 10.1109/BigMM50055.2020.00057 10.1109/BigMM50055.2020.00057

[3] : A Language-independent Network to Analyze the Impact of COVID-19 on the World via Sentiment Analysis. *ACM Transactions on Internet Technology* .2022;22(1) : 10.1145/3475867 1-30 10.1145/3475867

# Response

**Published:** 2022-05-13

**Authors:** Hu NG

**Affiliations:** Multimedia University, Malaysia

Dear Prof Yadav,

 We are greatly appreciative of the insightful comments and helpful suggestions that you have provided.

 The following are our response on the issues that you have highlighted:

 Your Comment

 1. The authors should give a more detailed description of the deep learning based methods used in sentiment analysis.

 Our Response

 Additional papers have been cited to provide more context on the deep learning based methods in the Literature Review. 

 Your Comment

 2. Also, they should tell how their proposed model is different from the other baseline models (apart from giving higher accuracy) as the area of sentiment classification have many popular models.

 Our Response

 This model places emphasis on attention mechanisms which in this context perform better than other baseline models. Remarks about this is added to the Methods.

 Your Comment

 3. Explain the role of attention mechanism in sentiment analysis. Can word-level and sentence level attention give better accuracy as explained in (A Language-independent Network to Analyze the Impact of COVID-19 on the World via Sentiment Analysis3)?

 Our Response

 The attention mechanism allows the model to utilize the most relevant parts of the input sequence in a flexible manner, by a weighted combination of all of the encoded input vectors, with the most relevant vectors being attributed the highest weights. We have not looked into sentence level attention as the focus of this paper is on word-level, and sentence-level will be explored in future work.

 Your Comment

 4. In the Introduction section, a bullet-wise summary of the significant contribution of this work is required, which could highlight the motivation for this study.

 Our Response

 A new paragraph has been added to highlight the contribution of this research work. We believe the paragraph format is more appropriate as follow the format of the journal 

 Your Comment

 5. There should be a Related work section in the manuscript which should explain how their proposed model differs from baseline methods or the advantages of their model over the baseline ones.

 Our Response

 The use of attention mechanisms in this context proves to be beneficial, and this is the advantage over baseline methods.

 Our Response

 New references added

 Yadav, A., & Vishwakarma, D. K. (2020). Sentiment analysis using deep learning architectures: a review. *Artificial Intelligence Review*, *53*(6), 4335-4385.

 Yadav, A., & Vishwakarma, D. K. (2020, September). A weighted text representation framework for sentiment analysis of medical drug reviews. In *2020 IEEE Sixth International Conference on Multimedia Big Data (BigMM)* (pp. 326-332). IEEE.

 Yadav, A., & Vishwakarma, D. K. (2021). A Language-independent Network to Analyze the Impact of COVID-19 on the World via Sentiment Analysis. *ACM Transactions on Internet Technology (TOIT)*, *22*(1), 1-30.
